# Evaluation of 90 day repeated dose oral toxicity and reproductive/developmental toxicity of 3'-hydroxypterostilbene in experimental animals

**DOI:** 10.1371/journal.pone.0172770

**Published:** 2017-03-03

**Authors:** Muhammed Majeed, Sarang Bani, Sankaran Natarajan, Anjali Pandey, Naveed S

**Affiliations:** 1 Sami Labs Limited, Bangalore, Karnataka, India; 2 Biological Research Department, Sami Labs Limited, Bangalore, Karnataka, India; 3 Research and Development Center, Sami Labs Limited, Bangalore, Karnataka, India; 4 ClinWorld, ClinWorld Private Limited, Bangalore, Karnataka, India; IIT Research Institute, UNITED STATES

## Abstract

3'-Hydroxypterostilbene (3'-HPT) is one of the active constituents of *Sphaerophysa salsula* and *Pterocarpus marsupium*. Despite many proposed therapeutic applications, the safety profile of 3'-HPT has not been established. The present work investigated 90 day repeated oral dose and reproductive (developmental) toxicity of 3'-HPT as a test substance in rats as per OECD guidelines. 90 day toxicity was conducted in sixty Sprague Dawley rats of each sex (120 rats), grouped into six dosage groups of 0 (control), 0 (control recovery), 20 (low dose), 80 (mid dose), 200 (high dose) and 200 (high dose recovery) mg/kg bwt/day (body weight/day) respectively. For the reproductive toxicity study forty Wistar rats of each sex (80 rats) divided into four dosage groups received 0 (vehicle control), 20 (low dose), 100 (mid dose) and 200 (high dose) mg/kg bwt/day of 3'-HPT respectively for a period of two weeks while pre-mating, mating, on the day before sacrifice, in females during pregnancy and four days of lactation period. Results showed no significant differences in body weight, food intake, absolute organ weight, haematology, with no adverse effects (toxicity) on biochemical values nor any abnormal clinical signs or behavioural changes were observed in any of the control/treatment groups, including reproductive and developmental parameters, gross and histopathological changes. In conclusion, the results suggested a No-Observed-Adverse-Effect-Level (NOAEL) of 200 mg/kg bwt/day in rats after oral administration, implying 3'-HPT did not exhibit any toxicity under the study conditions employed.

## Introduction

**Fig 1:**
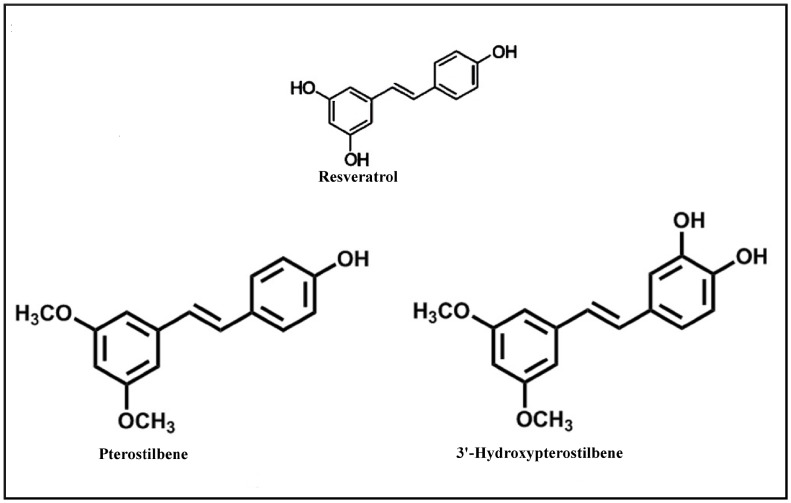
Stilbenoid analogues of resveratrol.

3'-Hydroxypterostilbene (3'-HPT) is a naturally occurring stilbenoid molecule present in the plants *Sphaerophysa salsula* [1], *Pterocarpus marsupium* and also in Honey Bee propolis (bee glue) [2]. *Sphaerophysa salsula* (Pall.) DC (Leguminosae) is a shrub widely distributed in the Central-Asia and in the northwest of China. It is used as a folk medicine for the treatment of hypertension in China [1]. 3'-HPT is an analogue of another natural stilbenoid pterostilbene which belongs to a class of compounds known as phytoalexins. Since pterostilbene is widely recognized for its anti-cancer and anti-diabetic properties, it is important to evaluate the toxicological role of its metabolites, as 3'-Hydroxypterostilbene has been found be one of the urinary metabolites of pterostilbene in mice [3]. Stilbene compounds like (trans-) resveratrol and piceatannol are widely distributed in nature. In view of their extensive range of biological activities such as cardioprotection and cancer chemopreventive properties, they have become of special interest to biologists and chemists. The trans-resveratrol-like natural stilbenoids have shown remarkable anti-cancer activity higher than resveratrol (Fig 1). 3'-HPT for example, is reported to be 50 to 97 times more potent than trans-resveratrol in eliciting significant apoptotic action on tumour cell lines and is markedly more active than pterostilbene in inducing apoptosis in sensitive and multi drug-resistant (MDR) leukemia cells [4]. Based on structure activity relationships, the importance of 3,5-dimethoxy motif in conferring pro-apoptic activity on leukemia cells has been recognized [5]. Pterostilbene and 3'-HPT possess the necessary 3,5-dimethoxy motif. Among these, 3'-HPT is an interesting natural compound which may be useful in treating different types of haematological malignancies as it is reported to possess a ‘privileged’ structure [4] motif of 3,5-dimethoxy group in addition to incorporating a piceatannol skeleton. 3'-HPT is a trans-stilbene molecule containing two methoxy groups in one ring and two hydroxy groups in the other ring. Recent research also revealed that 3'-HPT enhances the SIRT1 polypeptide activity and inhibits adipogenesis more effectively than resveratrol or its natural methoxylated derivative pterostilbene [6]. The antioxidant activity of 3'-HPT is approximately 30 times more potent than resveratrol, 70 times more than vitamin E, 80 times more than vitamin C and 90 times than BHT (butylated hydroxytoluene, the commonly used synthetic antioxidant) [7].

An important factor that hampers the utility of resveratrol as a dietary supplement is its questionable stability on storage [8]. In evaluating the structure-activity correlation, 3'-HPT possesses the skeleton structure of piceatannol, a known and active metabolite of resveratrol. The dimethoxy-substitution seems to confer relative stability on the molecule [9, 10]. 3'-HPT has been reported to be an excellent anti-proliferative and anti-apoptotic compound [5] and less toxic on normal haemopoietic stem cells than on leukemia and lymphoma cells [4]. In spite of the beneficial pharmacological and health promoting properties of 3'-HPT, there is a lacuna in information on its safety (toxicity) profile. Hence, the current study targeted at a 90 day repeated dose oral toxicity as well as a reproductive/developmental toxicity of 3'- HPT was performed.

## Materials and methods

### Chemicals and analysis of 3'-HPT

The chemicals and solvents used throughout the study were of analytical grade. The test item 3'- HPT was provided by Sami Labs Ltd., (Bengaluru, Karnataka, India). The 3'- HPT as provided was obtained through the steps disclosed in the US patent no. US 9458075 B1 [11].

The 3'-HPT as obtained had the following specifications and structural analysis was performed using IR spectrum, proton NMR, carbon NMR and mass spectrum. Analysis using HPLC indicated that the compound obtained was 98.31% w/w pure and was off white in color. Microbiological profile revealed negative for total yeast, *E*. *coli*, *Salmonella spp*., *Staphylococcus aureus*, *Pseudomonas aeruginosa and Enterobacteriaceae* when tested. Obtained compound was stable at 25 ± 2°C and 60 ± 5% RH conditions when tested for a period of three years. Compound was also found stable when tested at accelerated conditions 40 ± 2°C and 75 ± 5% RH for a period of 6 months. Its molecular formula, the molecular weight being C_16_H_16_O_4_ and 272.30 respectively, having the CA index name: 1,2-Benzenediol, 4-[(1E)-2-(3,5-dimethoxyphenyl)ethenyl]- (9CI).

### Animals

For reproductive/developmental toxicity study—young healthy adult Wistar rats aged 12–13 weeks at the time of mating as sourced from Dabur Research Foundation (DRF) animal facility and for 90 day repeated dose oral toxicity study- Sprague Dawley (SD) rats aged 6–8 weeks sourced from Bioneeds animal facility were used. These were housed in a controlled environment of temperature (22 ± 3°C) and relative humidity (RH) (50–60%), with photo periods of 12/12 h light-dark cycles. Provided with pelleted diet (Golden Feeds, New Delhi, India), filtered water *ad libitum*. Housing in cages: For reproductive/developmental toxicity study, five animals of same sex per cage before mating, two animals per cage of each sex during mating and pregnant females were individually housed with soft nesting material. For 90 day repeated dose oral toxicity study, two animals of same sex per cage were housed during acclimatization and study period.

### Ethics

Both 90 day repeated dose oral toxicity and reproductive/developmental toxicity studies were performed in strict accordance with the recommendation of the Committee for the Purpose of Control and Supervision of Experiments on Animals (CPCSEA) guidelines for laboratory animal facility, and as per OECD guideline 408, 421 respectively [12, 13]. The individual protocol was approved by the Institutional Animal Ethics Committee (IAEC) of Bioneeds (Registration No. 969/bc/06/CPCSEA) and Dabur Research Foundation (DRF) (Registration No.64/PO/br/s/99/CPCSEA) respectively. The animals were euthanized using carbon dioxide asphyxiation, and all efforts were made to minimize suffering.

### Dosing

The test item 3'- HPT was freshly prepared and daily administered orally after suspending it in corn oil (mg/kg/bwt).

### 90 day repeated dose oral toxicity study

#### Experimental design

The dose formulations, vehicle were administered to the treatment and recovery group animals (Table 1). Dose formulations, vehicle were administered by oral gavage once daily, to the respective groups, at the same time each day (varying by ± 2 h) for a period of 90 days with the actual volume calculated based on the recent body weight recorded at weekly intervals. There was no administration of test item formulations / vehicle during the 28-day recovery period in order to permit evaluation of the persistence, reversibility or delayed occurrence of toxic effects.

**Table 1:** Dosage groups for repeated dose 90 day oral toxicity study in SD rats.

Groups		Dose (mg/kg/d)	Concentration (mg/ml)	No. of Rats/group, Sex (n)	
				Male	Female
NG1	Vehicle Control (VC) (Corn oil)	0	NA	10	10
NG2	Control Recovery (CR) (Corn oil)	0	NA	10	10
NG3	Low dose (LD)	20	2	10	10
NG4	Mid dose (MD)	80	8	10	10
NG5	High dose (HD)	200	20	10	10
NG6	High dose Recovery (HDR)	200	20	10	10

NA- Not Applicable; n = number.

#### Clinical observations

All treated, control rats were observed twice (morning and afternoon) daily for clinical signs and mortality for a total period of 90 days. The body weight of each rat was recorded prior to treatment on day 1, weekly thereafter and at end of the 90 day study. Group mean body weights were calculated. The food consumption of all rats in each cage was recorded from day of commencement of treatment and average weekly consumption was calculated. During post-treatment period, food consumption was recorded weekly for recovery group rats.

#### Neurobehavioural assessment

The neurological examination was carried out on all the animals in twelfth week of the study. The neurobehavioural assessment (functional examination) of sensory reactivity to different types of auditory, visual and proprioceptive stimuli (landing foot splay, righting reflex, and gait), assessment of grip strength and motor activity assessment were recorded (Table 2). At the end of treatment all surviving animal were fasted overnight with water given *ad libitum*.

**Table 2:** Effect of 90 day oral administration of 3'-HPT on functional observation battery (neurobehavioural assessment) tests in male and female SD rats.

Groups	Functional observation battery										Neurobehavioural response	
	Dose (mg/kg/d)	Visual Response	Auditory Response	Response to Proprioceptive Stimuli								
				Gait	Landing Foot splay (cm)		Righting Reflex					
							a	b	c	d	Locomotor Activity	Grasping strength
					Male	Female						
NG1 (VC)	0	0	0	0	3.4 ± 0.9	3.8 ± 0.9	0	0	0	0	0	0
NG2 (CR)	0	0	0	0	3.9 ± 0.7	4.3 ± 1.2	0	0	0	0	0	0
NG3 (LD)	20	0	0	0	3.6 ± 1.0	3.6 ± 0.9	0	0	0	0	0	0
NG4 (MD)	80	0	0	0	3.5 ± 0.8	4.0 ± 0.8	0	0	0	0	0	0
NG5 (HD)	200	0	0	0	4.1 ± 0.9	4.1 ± 0.9	0	0	0	0	0	0
NG6 (HDR)	200	0	0	0	3.5 ± 0.6	3.9 ± 1.0	0	0	0	0	0	0

n = 10; 0: Normal; a: on back; b: body tilted; c: head tilted; d: dropped. Values are in Mean ± SEM.

#### Ophthalmological examination

Ophthalmological examination was carried out at the beginning and at termination of the study for all the groups.

#### Haemotology and clinical chemistry

Blood samples were collected in EDTA tubes for determination of haematological parameters (Table 3) and heparin tubes for clinical chemistry (Table 4).

**Table 3:** Effect of 90 day oral administration of 3'-HPT on haematology data of male and female SD rats.

Parameters	Groups / Dose (mg/kg/d)	NG1 (VC) / (0)	NG2 (CR) / (0)	NG3 (LD) / (20)	NG4 (MD) / (80)	NG5 (HD) / (200)	NG6 (HDR) / (200)
	Sex						
Total Leucocyte Count (10^3^ cells/μl)	M	14.9 ± 2.8	15.6 ± 3.2	14.4 ± 3.1	14.9 ± 2.3	15.5 ± 2.7	17.7 ± 1.5
	F	10.0 ± 2.9	9.5 ± 2.0	8.5 ± 1.3	8.7 ± 2.5	11.2 ± 2.9	9.1 ± 2.8
Total Erythrocyte count (10^6^ cells/μl)	M	7.5 ± 0.3	7.5 ± 0.4	7.5 ± 0.4	7.3 ± 0.8	7.7 ± 0.5	8.0 ± 0.6
	F	6.6 ± 0.4	7.0 ± 0.8	7.0 ± 0.6	6.6 ± 0.6	6.8 ± 0.7	6.7 ± 0.6
Haemoglobin (g/dl)	M	13.2 ± 0.6	13.1 ± 0.6	13.2 ± 0.8	13.0 ± 0.5	12.9 ± 0.7	13.7 ± 1.0
	F	12.4 ± 0.8	13.0 ± 1.2	13.1 ± 1.2	12.5 ± 1.4	12.8 ± 1.2	12.6 ± 0.8
Haematocrit (%)	M	42.9 ± 1.9	42.5 ± 2.2	42.9 ± 2.3	41.1 ± 4.9	42.7 ± 2.7	44.5 ± 3.2
	F	38.1 ± 2.2	39.8 ± 3.8	39.9 ± 3.2	38.0 ± 4.1	38.8 ± 3.6	38.4 ± 2.2
Mean corpuscular volume (fL)	M	57.4 ± 2.1	56.4 ± 1.9	57.0 ± 1.6	55.9 ± 0.9	55.6 ± 1.9	55.8 ± 2.4
	F	57.6 ± 1.3	56.6 ± 1.6	57.0 ± 2.0	57.1 ± 2.3	57 ± 2.1	57.2 ± 3.4
Mean corpuscular haemoglobin (pg)	M	17.8 ± 0.6	17.4 ± 0.5	17.6 ± 0.8	17.9 ± 2.9	16.9 ± 0.6	17.1 ± 0.8
	F	18.8 ± 0.8	18.6 ± 0.9	18.8 ± 1.1	18.7 ± 0.9	18.8 ± 0.8	18.8 ± 1.2
Mean corpuscular haemoglobin concentration (g/dl)	M	30.9 ± 0.4	30.9 ± 0.4	30.8 ± 1.1	32.1 ± 5.6	30.3 ± 0.4	30.7 ± 0.9
	F	32.6 ± 0.7	32.8 ± 0.2	33.0 ± 1.2	32.8 ± 0.4	32.9 ± 0.6	32.9 ± 0.7
Platelet Count (10^3^ cells/μl)	M	688.7 ± 123.7	624.0 ± 85.5	633.5 ± 100.1	618.3 ± 169.6	714.4 ± 83.8	703.8 ± 61.9
	F	641.2 ± 81.3	659.6 ± 109.4	621.8 ± 81.3	563.6 ± 144.6	639.5 ± 96.0	593.8 ± 120.4
Blood clotting time (Sec)	M	72.9 ± 16.9	70.3 ± 14.3	66.7 ± 8.3	67.1 ± 18.2	60.7 ± 13.6	67.9 ± 15.7
	F	55.8 ± 22.5	62.9 ± 18.8	51.0 ± 11.5	53.5 ± 14.5	57.2 ± 18.2	59.4 ± 11.2
Neutrophils (%)	M	15.6 ± 5.5	20.6 ± 7.0	19.2 ± 5.4	16.1 ± 4.7	17.2 ± 4.8	17.3 ± 5.9
	F	20.4 ± 4.9	21.4 ± 5.1	17.6 ± 6.3	20.7 ± 5.8	20.9 ± 6.6	23.3 ± 6.1
Lymphocytes (%)	M	84.2 ± 5.8	81.0 ± 6.3	80.7 ± 5.4	83.7 ± 5.0	82.4 ± 5.2	82.6 ± 5.9
	F	79.4 ± 4.7	78.2 ± 5.5	82.0 ± 6.6	79.0 ± 6.2	78.8 ± 6.8	76.6 ± 6.1
Eosinophils (%)	M	0.1 ± 0.3	0.0 ± 0.0	0.0 ± 0.0	0.0 ± 0.0	0.1 ± 0.3	0.1 ± 0.3
	F	0.0 ± 0.0	0.1 ± 0.3	0.1 ± 0.3	0.3 ± 0.5	0.0 ± 0.0	0.0 ± 0.0
Monocytes (%)	M	0.1 ± 0.3	0.1 ± 0.3	0.1 ± 0.3	0.2 ± 0.6	0.3 ± 0.7	0.0 ± 0.0
	F	0.2 ± 0.6	0.3 ± 0.7	0.3 ± 0.7	0.2 ± 0.6	0.3 ± 0.7	0.1 ± 0.3

n = 10; M, male; F, female. Values are in Mean ± SD.

**Table 4:** Effect of 90 day oral administration of 3'-HPT on biochemical data of male and female SD rats.

Parameter	Groups / Dose (mg/kg/d)	NG1 (VC) / (0)	NG2 (CR) / (0)	NG3 (LD) / (20)	NG4 (MD) / (80)	NG5 (HD) / (200)	NG6 (HDR) / (200)
	Sex						
Glucose (mg/dl)	M	112.70 ± 18.18	109.40 ± 18.90	105.70 ± 15.09	100.39 ± 16.39	101.80 ± 12.84	98.70 ± 12.28
	F	107.40 ± 18.65	114.50 ± 14.18	106.90 ± 10.80	105.10 ± 19.93	99.00 ± 13.78	103.10 ± 12.27
Total Cholesterol (mg/dl)	M	42.10 ± 6.47	40.20 ± 7.94	37.80 ± 8.99	43.00 ± 9.76	38.30 ± 9.51	41.10 ± 7.71
	F	57.50 ± 12.84	58.10 ± 8.52	50.70 ± 6.57	48.00 ± 8.94	47.40 ± 9.49	48.50 ± 7.81
Creatinine (mg/dl)	M	0.86 ± 0.15	0.78 ± 0.04	0.77 ± 0.07	0.82 ± 0.09	0.77 ± 0.12	0.78 ± 0.14
	F	0.98 ± 0.06	0.99 ± 0.03	0.98 ± 0.13	0.97 ± 0.18	0.93 ± 0.13	0.96 ± 0.08
Total Protein (mg/dl)	M	5.62 ± 0.88	5.62 ± 0.38	5.55 ± 0.25	5.28 ± 0.44	5.64 ± 0.52	5.69 ± 0.37
	F	6.85 ± 0.35	6.99 ± 0.43	6.78 ± 0.43	7.01 ± 0.52	7.15 ± 0.39	7.00 ± 0.49
Albumin (mg/dl)	M	3.51 ± 0.22	3.31 ± 0.17	3.28 ± 0.25	3.28 ± 0.19	3.38 ± 0.17	3.47 ± 0.28
	F	3.90 ± 0.27	3.93 ± 0.47	3.89 ± 0.23	4.12 ± 0.27	4.03 ± 0.18	3.96 ± 0.36
Triglycerides (mg/dl)	M	60.05 ± 13.71	68.70 ± 14.00	65.00 ± 14.41	69.40 ± 20.66	50.40 ± 12.37	52.00 ± 12.34
	F	69.30 ± 14.85	71.70 ± 21.15	55.90 ± 20.37	59.40 ± 13.33	55.90 ± 21.12	52.50 ± 16.22
Urea Nitrogen (mg/dl)	M	13.42 ± 2.42	14.64 ± 1.65	14.62 ± 2.76	15.35 ± 3.24	16.03 ± 1.90	16.01 ± 1.89
	F	18.33 ± 4.23	19.91 ± 5.21	15.75 ± 2.51	15.60 ± 2.29	17.54 ± 2.50	19.27 ± 3.79
Total bilirubin (mg/dl)	M	0.33 ± 0.08	0.36 ± 0.07	0.33 ± 0.07	0.30 ± 0.08	0.32 ± 0.09	0.29 ± 0.07
	F	0.37 ± 0.13	0.39 ± 0.12	0.32 ± 0.04	0.35 ± 0.07	0.33 ± 0.11	0.30 ± 0.05
Alanine aminotransferase (U/L)	M	77.50 ± 20.46	67.60 ± 19.08	60.30 ± 14.65	61.20 ± 12.78	54.20*± 18.08	53.30*± 10.63
	F	73.00 ± 16.15	78.00 ± 14.73	74.40 ± 18.07	65.30 ± 10.06	56.10*± 10.65	57.40*± 7.83
Aspartate aminotransferase (U/L)	M	166.60 ± 17.52	168.40 ± 15.09	163.90 ± 25.05	149.00 ± 23.49	139.30*± 9.17	140.00*± 12.82
	F	172.70 ± 20.34	169.40 ± 16.71	171.60 ± 17.76	167.30 ± 19.45	147.50*± 20.64	143.20*± 21.47
High density lipoprotein (mg/dl)	M	22.67 ± 1.95	22.56 ± 3.64	21.00 ± 5.49	24.07 ± 3.50	21.37 ± 4.80	27.43 ± 5.01
	F	27.85 ± 6.20	26.63 ± 4.87	26.60 ± 4.58	27.31 ± 4.80	30.82 ± 6.10	31.59 ± 4.69
Alkaline phosphatase (U/L)	M	143.10 ± 18.79	152.70 ± 22.07	151.80 ± 25.68	170.66 ± 23.42	144.70 ± 33.46	160.15 ± 21.45
	F	144.44 ± 19.94	139.84 ± 27.01	145.68 ± 29.32	154.78 ± 31.17	163.13 ± 19.77	159.60 ± 22.95
Low density lipoprotein (mg/dl)	M	17.26 ± 2.37	13.86 ± 2.29	14.95 ± 3.80	16.47 ± 2.39	19.94 ± 4.67	17.69 ± 3.54
	F	16.36 ± 3.03	14.95 ± 7.12	15.60 ± 3.30	15.00 ± 3.40	12.14 ± 2.10	14.55 ± 3.16
Calcium (mg/dl)	M	9.26 ± 0.77	8.77 ± 1.12	9.09 ± 0.90	9.18 ± 0.86	9.09 ± 0.90	8.87 ± 0.96
	F	9.14 ± 0.71	8.91 ± 1.09	17.51 ± 26.89	8.92 ± 0.97	8.79 ± 0.84	9.31 ± 1.12
Phosphorous (mg/dl)	M	3.97 ± 0.62	3.75 ± 0.56	3.67 ± 0.57	4.94 ± 0.90	4.66 ± 1.40	4.55 ± 1.64
	F	4.19 ± 0.59	4.14 ± 0.32	4.31 ± 0.51	4.20 ± 0.69	4.76 ± 0.58	4.72 ± 0.93
Sodium (mmol/L)	M	141.40 ± 4.38	140.60 ± 4.67	140.20 ± 3.19	139.20 ± 7.11	140.80 ± 1.93	140.60 ± 4.72
	F	141.30 ± 2.67	139.80 ± 4.87	140.90 ± 3.73	140.80 ± 3.74	141.50 ± 3.63	141.60 ± 2.59
Potassium (mg/dl)	M	4.28 ± 0.57	4.18 ± 0.70	4.14 ± 0.67	4.11 ± 0.28	3.94 ± 0.39	4.10 ± 0.34
	F	4.19 ± 0.34	4.11 ± 0.52	4.18 ± 0.47	4.16 ± 0.68	4.11 ± 0.28	4.06 ± 0.43

n = 10; M, male; F, female. Values are in Mean ± SD;

* Statistically significant (P<0.05) compared with the VC group.

#### Necropsy/Gross pathology and organ weights

On completion of 90 days and 118 days in case of treatment and recovery periods respectively, all surviving rats were fasted overnight, subjected to complete necropsy, weighed before exsanguination, and gross pathological examination was performed. The organs thus collected (Table 5) from all animals were trimmed of adherent tissue, fat as appropriate and weighed wet as soon as possible to avoid drying.

**Table 5:** Effect of 90 day oral administration of 3'-HPT on absolute organ weight (mg) of male and female SD rats.

Organs	Groups/ Dose (mg/kg/d)	NG1 (VC) / (0)	NG2 (CR) / (0)	NG3 (LD) / (20)	NG4 (MD) / (80)	NG5 (HD) / (200)	NG6 (HDR) / (200)
	Sex						
Liver	M	8751.4 ± 1070.6	9867.3 ±1420.2	8931.7 ± 1279.6	9008.1 ± 1295.5	7630.4 ± 3601.3	7998.8 ± 1561.2
	F	6817.0 ± 714.1	6701.5 ± 1258.3	6529.3 ± 663.9	6908.9 ± 386.7	6891.1 ± 383.8	7217.3 ± 1138.9
Kidneys	M	1986.9 ± 317.4	2422.9 ± 383.4	1766.6 ± 617.8	1893.5 ± 242.1	1869.2 ± 198.8	2088.2 ± 288.1
	F	1447.3 ± 175.6	1493.7 ± 173.6	1294.7 ± 160.2	1437.7 ± 94.5	1449.1 ± 157.6	1515.6 ± 198.4
Adrenals	M	43.5 ± 8.7	53.1 ± 6.8	49.4 ± 11.0	49.0 ± 9.3	52.1 ± 14.6	54.4 ± 7.7
	F	64.9 ± 8.9	64.0 ± 14.2	64.9 ± 13.4	67.3 ± 16.2	68.2 ± 8.1	65.9 ± 14.9
Testes / Ovaries	M	2951.2 ± 343.0	3095.5 ± 375.3	3060.6 ± 359.0	3125.8 ± 316.1	2918.9 ± 248.2	2940.6 ± 543.8
	F	101.4 ± 41.0	107.0 ± 18.3	94.5 ± 14.7	101.0 ± 20.5	85.1 ± 17.3	107.4 ± 18.8
Epididymides / Uterus	M	1302.1 ± 160.9	1393.2 ± 235.6	1179.6 ± 117.0	1189.0 ± 211.2	1208.1 ± 147.4	1331.6 ± 245.0
	F	1156.1 ± 156.2	1122.2 ± 255.7	1119.9 ± 194.9	1149.4 ± 114.0	1109.5 ± 254.5	1082.7 ± 194.2
Thymus	M	294.8 ± 72.3	363.9 ± 42.0	317.2 ± 61.5	260.7 ± 96.4	277.3 ± 102.7	385.7 ± 89.6
	F	365.7 ± 91.1	301.6 ± 81.0	360.9 ± 76.3	309.5 ± 62.3	339.4 ± 60.0	272.9 ± 72.5
Spleen	M	1141.1 ± 163.5	1092.2 ± 128.5	1154.7 ± 222.9	1003.7 ± 158.1	917.9 ± 120.6	956.9 ± 148.0
	F	928.3 ± 98.9	864.7 ± 152.1	790.3 ± 81.6	901.7 ± 181.2	917.6 ± 188.2	844.2 ± 188.0
Brain	M	1844.1 ± 171.0	1986.3 ± 88.8	1869.0 ± 102.5	1859.7 ± 179.1	1810.6 ± 109.0	1920.2 ± 170.3
	F	1862.4 ± 104.6	1817.7 ± 103.8	1826.3 ± 108.9	1819.3 ± 161.6	1885.4 ± 92.5	1839.8 ± 132.1
Heart	M	1010.0 ± 154.1	1174.0 ± 117.7	990.6 ± 130.5	973.2 ± 136.0	926.3 ± 102.8	1103.2 ± 130.3
	F	831.5 ± 63.4	814.4 ± 128.2	812.4 ± 99.8	845.1 ± 60.0	833.8 ± 114.7	795.2 ± 64.1

n = 10; M, male; F, female; values in Mean ± S.D.

#### Histopathology

Histopathological examination was performed on tissues of all animals from the control and the high dosage group, sacrificed at scheduled termination. The tissues / organs (Mesenteric lymph node, thymus, trachea, lungs, heart, thyroid, stomach, small intestine, large intestine (colon), liver, spleen, kidneys, adrenals, epididymides / uterus, testes / ovaries and brain) from all animals, were collected and preserved in 10% neutral buffered formalin with the eyes preserved in Davidson’s fluid.

### Reproductive/Developmental toxicity study

#### Experimental design

The animal groups (Table 6) of each sex were dosed once daily for a period of two weeks while pre-mating, mating, on the day before sacrifice and in females during pregnancy and four days of lactation period. Mating ratio of 1:1 (one male to one female), was allowed by housing them in single cages, subsequently the female animals were caged individually upon confirmation of pregnancy.

**Table 6:** Dosage groups for reproduction/developmental toxicity study in Wistar rats.

Groups		Dose (mg/kg/d)	Concentration (mg/ml)	No. of Rats/group, Sex (n)	
				Male	Female
RG1	Vehicle Control (VC) (Corn oil)	0	NA	10	10
RG2	Low dose (LD)	20	2	10	10
RG3	Mid dose (MD)	100	10	10	10
RG4	High dose (HD)	200	20	10	10

NA- Not Applicable; n = number.

#### Clinical observations

All animals were observed once daily throughout the period of study for clinical signs of toxicity. Rats were observed for clinical signs like changes in skin and fur, eyes, breathing, mucous membrane, occurrence of secretions and excretions, changes in gait, posture and presence of any abnormal behaviour (data not included). All animals were weighed on the first day of dosing, subsequently weekly once and at study termination. During pregnancy, females were weighed on day 0, 7, 14 and 21, 24 h of parturition and on day 4 post partum. Pups which were alive were counted, sexed and weighed within 24 hours of parturition and on day 4 post partum. The quantity of food consumed by the animals (g/rat/day (d)) of the treatment group was measured during in-life phase (pre-mating, pregnancy and lactation) of the study. The food consumed was recorded for each animal per dose group on weekly basis.

#### Necropsy, gross pathology, organ weights and histopathology

The rats to be sacrificed at term (males on day 32 and females on day 54) were anaesthetized, weighed and exsanguinated. During necropsy, the animals were examined visually (macroscopically) for any abnormalities or pathological changes. The organs showing macroscopic lesions were preserved and further subjected to microscopic examination. Special attention was paid to the reproductive organs to detect abnormalities, if any. The number of corpora lutea and implantation sites were recorded. The reproductive organs were removed (ovaries, testes, uterus and epididymides), examined, the weight of these organs were recorded as absolute values and subsequently placed in Bouin’s fixative for histopathological examination. Histopathological and gross lesions examination was carried out on the preserved tissues of vehicle control (RG1) and high dose (RG4) group.

### Statistical analysis

The data (body weight, food intake, organ weights, haematology and clinical chemistry) generated from the 90 day repeated dose oral toxicity and reproductive/developmental toxicity study was subjected to statistical analysis using GraphPad prism version 5.00 (2007), GraphPad Software, USA. The statistical analysis of the 90 day repeated dose oral toxicity experimental data was carried out using one way ANOVA with Dunnet’s post test done for different treatment groups comparing with the control group data. The unpaired ‘t’-test was done for control recovery and high dose recovery group data. All analysis and comparisons were evaluated at 5% (P<0.05) level. The statistical analysis of the reproductive/developmental toxicity experimental data was carried out for differences among treated / control groups using one way ANOVA.

## Results

### 90 day repeated oral toxicity

The 90 day repeated oral toxicity in SD rats was evaluated with the 3'-HPT being administered orally in corn oil (vehicle) to the treatment group animals at different doses per kg bwt/day except control group (NG1) and control recovery (NG2) groups (Table 1). The vehicle and 3'-HPT administration to control recovery and high dose recovery (NG6) group animals respectively was stopped after 90 days of administration and observed for another 28 days to evaluate the effects during recovery period.

#### Changes in body weight and food consumption

**Fig 2:**
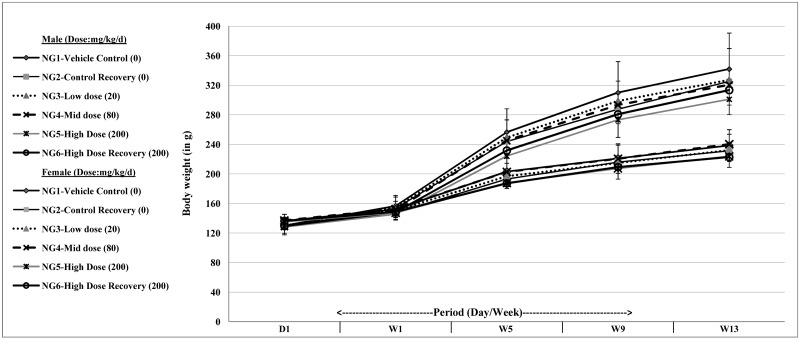
Body weight changes on oral administration of 3'-HPT measured over 13 weeks (90 days) in male and female SD rats. D1: Day one; W1, W5, W9 and W13: Week one, five, nine and thirteen. Data in Mean ± SD (n = 10/group).

The body weights of the rats were monitored from day one till the end of thirteen weeks (90 days) (Fig 2). At the end of first week the mean body weights of both male and female rats were found to have increased following treatment. The final mean body weights in the male rats were higher than that in the female rats. The overall changes in body weight among the groups within both male and female rats were found to be not statistically significant. Food consumption was monitored from the first week for all groups NG1 to NG6 till the end of thirteen weeks (90 days) and was found to be comparable across all the test groups (data presented in S1 Fig). A slight decrease (not statistical significant) in the mean average food consumption was observed in the high dose group (NG5: 7.96 ± 1.70 g/rat/day) as compared to the vehicle control group (NG1: 11.16 ± 1.59 g/rat/day) at the end of first week in males (S1A Fig). A similar trend (not statistically significant) was observed in females (S1B Fig), in the mid dose group (NG4) with the mean average food consumption being 6.93 ± 0.99 g/rat/day at end of thirteenth week compared within the group at 9.09 ± 0.67 g/rat/day at end of first week, but the thirteenth week value of NG4 group was comparable to the value of vehicle control (NG1: 7.00 ± 0.51 g/rat/day) at the end of week thirteen. However, no significant differences in mean average food consumption were observed in both males and females in any treatment groups compared to that of the respective control groups.

#### Neurobehavioural assessment

Functional observational battery tests such as visual response, auditory response, gait, landing foot splay, righting reflex including grasping strength and locomotor activity measures performed (Table 2) on all test item treated group rats up to the highest dose (200 mg/kg bwt/d) did not reveal any treatment related abnormalities.

#### Ophthalmic examinations

No treatment-related ophthalmic findings were observed in control animals or treatment animals of both sexes (data not included).

#### Haematology and clinical chemistry

The haematological data (Table 3) of the control and treated rats on repeated oral treatment for 90 days did not cause any significant changes in any of the haematological parameters at all the doses tested. However, in clinical chemistry a significantly lower (P<0.05) Alanine aminotransferase (ALT) and Aspartate aminotransferase (AST) in high dose (NG5) and high dose recovery (NG6), in both male and female groups during treatment was observed with all the other biochemical parameters remaining unaffected (Table 4).

#### Necropsy/Gross pathology, organ weights and histopathology

No gross lesions during necropsy were observed in any of the animals in treatment and recovery groups, indicating that the test item did not cause any lesions systemically (data not included). None of the animals in treatment and recovery groups showed any variations in mean value of the absolute organ weights (Table 5) compared to respective control groups indicating that the test item did not had any adverse effects on organ weights. None of the animals in any of the groups showed any histological changes during evaluation, indicating that the test item did not cause any histological changes in the tissues (data not included).

### Reproductive/Developmental toxicity study

To evaluate the reproductive/developmental toxicity, control animals were administered only with corn oil, while the other treatment groups were administered with the test item i.e. 3'-HPT at different dosages per kg bwt/d (Table 6).

#### Body weight changes and food consumption

Males and females (during pre-mating period) exhibited an increase in body weight during the study period with the female body weight not being affected by the administration of 3'-HPT during the gestation and lactation period and was comparable with the control group (data not included). The changes in food consumption (g/rat/d) of both sexes during pre-mating was not statistically significant and comparable to the control group and that of the females (g/rat/d) during the gestation, lactation period was comparable to the control group (data not included). Mean body weight of both male and female pups remained unaffected during lactation period (Table 7).

**Table 7:** Body weight of male and female pups (in g) during lactation in reproductive/developmental toxicity study of 3'-HPT in Wistar rats.

Group	Dose (mg/kg/d)	Lactation period			
		Day 1		Day 4	
		Male	Female	Male	Female
RG1 (VC)	0	27.8 ± 4.02	31.0 ± 1.93	36.1 ± 4.90	37.2 ± 3.40
RG2 (LD)	20	25.8 ± 3.04	30.7 ± 2.74	29.5 ± 5.44	37.7 ± 6.11
RG3 (MD)	100	21.8 ± 4.15	23.1 ± 5.32	29.4 ± 7.51	30.3 ± 8.08
RG4 (HD)	200	38.7 ± 6.33	24.7 ± 3.45	40.9 ± 8.00	26.7 ± 4.71

Values are in Mean ± SEM.

#### Necropsy/Gross pathology, organ weights and histopathology

The animals were evaluated via gross visual observation, the male and female genitals and other organs with gross lesions were subjected to microscopic examination. Histopathology of the tissues of control and high dose groups did not show any test item related abnormality across all groups. The ovaries and epididymides did not show any test item related abnormality. However, the testes from the high dose group showed a mild increase in the number of seminiferous tubules with a mean value of 1.6% as against the control mean value of 0.3%. One uterus from the high dose group showed pronounced infiltration of endometrial tissues by eosinophils. The absolute organ weight of males (testes and epididymides) and females (ovaries and uterus) were not affected and found to be comparable with the control group (Table 8) after oral exposure to 3'-HPT. The mean number of implantations, mean number of corpora lutea (Table 9), number of pregnancies, number of dams littered, gestation length and number of days of pregnancy (Table 10) remained unaffected by the daily oral exposure of 3'-HPT. Females across groups showed normal pregnancy (in days). No dose dependent changes were observed in pre-coital interval, percentage of pre and post-implantation loss in all treatment groups (Table 10).

**Table 8:** Absolute organ weights (in g) of animals upon 3'-HPT administration in reproductive/ developmental toxicity study of 3'-HPT in Wistar rats.

Group	Dose (mg/kg/d)	Male		Female	
		Testes	Epididymides	Ovaries	Uterus
RG1 (VC)	0	3.03 ± 0.09	1.19 ± 0.06	0.17 ± 0.01	0.60 ± 0.04
RG2 (LD)	20	2.9 ± 0.1	1.21 ± 0.04	0.18 ± 0.01	0.62 ± 0.04
RG3 (MD)	100	3.0 ± 0.13	1.24 ± 0.05	0.17 ± 0.01	0.65 ± 0.07
RG4 (HD)	200	3.1 ± 0.08	1.3 ± 0.04	0.17 ± 0.00	0.58 ± 0.04

Values are in Mean ± SEM.

**Table 9:** Corpora lutea and implantation sites in numbers in reproductive/ developmental toxicity study in Wistar rats.

Group	Dose (mg/kg/d)	No. of Corpora lutea (CL)	No. of implantations (IMP)	Successful implantations (%)
		Total (Mean)	Total (Mean)	
RG1 (VC)	0	11.63	11.63	100
RG2 (LD)	20	11.14	10.29	92.31
RG3 (MD)	100	10.57	10.86	102.7
RG4 (HD)	200	12.00	11.00	91.67

**Table 10:** Summary of effects of 3'-HPT administration on reproduction/development in Wistar rats.

Groups / Dose (mg/kg/d)	VC (RG1) / (0)	LD (RG2) / (20)	MD (RG3) / (100)	HD (RG4) / (200)
Observations				
Pairs started (N)	10	10	10	10
Females showing evidence of copulation (N)	10	09	09	10
Females achieving pregnancy (confirmed at necropsy) (N)	08	08	07	07
Pre-coital interval^$^ (Days)	4.6 ± 0.43	3.11 ± 0.57	3.67 ± 2.49	4.20 ± 3.31
Gestation length^$^ (Days)	22 ± 0.43	22.14 ± 0.35	22 ± 0.00	21.14 ± 0.35
Pregnancy ≤ 21 days (N)	0	0	0	0
Pregnancy = 22 days (N)	06	06	07	06
Pregnancy ≥ 23 days (N)	02	01	0	01
Dams littered (N)	08	07	07	07
Dams dead at gestation (N)	0	01	0	0
Dams dead at Lactation (N)	0	0	0	0
Dams with total litter loss on day 4 of lactation (N)	0	01	01	0
Corpora lutea/Group (mean)	11.63	11.14	10.57	12.00
No. of Implantations/Group (mean)	11.63	10.29	10.86	11.00
Live pups/dam at birth (mean)	10.75	10	9.43	9.71
Live pups/dam at day 4 (mean)	10.13	09	07	9.14
Sex ratio (M:F) at birth	0.85	0.79	0.89	1.31
No. of live pups (M+F) at day 4	81	63	49	64
Pup weight at day 1^$ $^ (M)	27.8 ± 4.02	25.8 ± 3.04	21.8 ± 4.15	38.7 ± 6.33
Pup weight at day 1^$ $^ (F)	31.0 ± 1.93	30.7 ± 2.74	23.1 ± 5.32	24.7 ± 3.45
Pup weight at day 4^$ $^ (M)	36.1 ± 4.9	29.5 ± 5.44	29.4 ± 7.51	40.9 ± 8.00
Pup weight at day 4^$ $^ (F)	37.2 ± 3.4	37.7 ± 6.11	30.3 ± 8.08	26.7 ± 4.71
No. of pups dead at birth (N)	0	0	02	06
Pre-implantation loss (%)	0	7.69	-2.7	8.33
Post-implantation loss (%)	6.45	2.78	13.16	10.39

N = number; M, male; F, female; Pup weight (in g).

^$^ Values in Mean ± SD;

^$ $^ Values in Mean ± SEM.

## Discussion

3'-HPT an analogue of resveratrol is found naturally in plants such as *Sphaerophysa salsula* [1], *Pterocarpus marsupium*, and Honey Bee propolis (bee glue) [2]. The health benefits of stilbenoid compounds such as resveratrol are well established. Various pharmacological activities have since been associated with this compound and its close analogues (pterostilbene and 3'-HPT) have been the subject of numerous studies [2]. However, 3'-HPT was found markedly more active than the above compounds, displaying a low toxicity on normal haemopoietic stem cells [4]. 3'-HPT has been evidenced to have anti-adipogenic, anti-inflammatory, anti-oxidant, Histone Deacetylase (HDAC), and Sirtuin 1 (SIRT1) inhibitory activity. Collectively, the increased anti-oxidant, anti-adipogenic activity, cell selectivity and COX-2 specificity of 3'-HPT was found in support of its activity in preventing cardiovascular and pulmonary diseases. Coupled with HDAC and SIRT1 inhibitory activity, 3'-HPT has been found to demonstrate protective effects from neurodegenerative diseases and select cancers [14].

In spite of the several beneficial and therapeutic properties proposed, studies evaluating the toxicity of the 3'-HPT have not been reported. Hence the current study was proposed.

In current 90 day repeated oral toxicity study, all the animals were observed for health status, clinical signs of toxicity and mortality; weekly detailed veterinary examination (data not included) and subjected to neurological assessments. Body weight changes observed in both the sexes at 200 mg/kg bwt/d especially in the males (NG5) during the first week was considered as non adverse as this was not a common observation in other groups. Also the slight decrease in the food consumption observed in the high dose group (NG5) at the end of first week in males and in the mid dose group (NG4) in females at the end of thirteenth week was not statistically significant for both males and females and was comparable across the groups in the subsequent weeks in case of males, and in case of females comparable across other treatment groups including vehicle control group (NG1) inferring that the 3'-HPT treatment did have any statistically significant effect on the food consumption in rats and was unrelated to body weight of both sex. The functional observational battery tests related to the neurobehavioral assessment were not affected as no treatment related abnormalities were observed even at the highest dose (NG5) of 200 mg/kg bwt/d. The same was true with the ophthalmic examinations with no changes observed in all the groups. The haematological data was comparable at all doses tested with no significant changes observed in other clinical chemistry parameters estimated in all the groups. The lower values observed in the Alanine aminotransferase (ALT) and Aspartate aminotransferase (AST) in high dose (NG5) and high dose recovery (NG6), in both male and female groups during treatment were shown to be of non-pathological importance, however, there were no changes observed in the AST and ALT concentrations at the end of recovery period, hence considered to be non adverse as the decrease in serum AST and ALT concentrations has not been shown to be a pathologically important phenomenon which was unlikely to be caused by the oral exposure to 3'-HPT. Necropsy, gross pathology and histopathological examination of tissues and organs did not reveal any pathological changes. In treatment and recovery groups, the mean absolute organ weight of male and female rats at necropsy observed for liver, kidneys, brain, spleen, heart, thymus testes/ovaries, epididymides/uterus, and adrenals, did not show any statistically significant variations in absolute organ weights suggesting that the test item did not have any evident toxicological effects upon administration. The reported results showed that the No-Observed-Adverse-Effect-Level (NOAEL) of 3'-hydroxypterostilbene in SD rats, following repeated 90 day oral route administration was found to be 200 mg/kg bwt.

In reproductive/developmental toxicity study the animal groups of each sex were dosed for a period of two weeks while pre-mating, mating, on the day before sacrifice and in females during gestation and four days of lactation period. Increase in the body weight of male and female rats during pre-mating period and the comparable weights of the female body weights with the control group during the gestation period, the food consumption being comparable among both sexes during pre-mating and in females during gestation to control groups, with the mean body weights of the male and female pups remaining unaffected during lactation period suggested the absence of any adverse effects or dose associated changes on the body weight, food consumption, mean body weight of pups following repeated oral dosing of 3'-HPT.

The histopathology of the tissues (genitals) of all high dose (RG4) and control (RG1) groups lacked any abnormalities owing to the exposure to the test item. A mild increase in the mean value of the number of seminiferous tubules of the testes in high dose group (RG4) compared to the controls (RG1) was not a common observation in other dosage groups; infiltration of endometrial tissues in one of the high dose group observed indicated an allergic reaction. However, as there was no change in the absolute organ weight of males (testes and epididymides) and females (ovaries and uterus) which indicated that the findings were incidental and not dose dependent, suggesting that the changes were not toxicologically relevant to the test item. The lack of maternal toxicity with no changes in number of pregnancies, number of dams littered, gestation length, number of days of pregnancy, mean number of implantations and corpora lutea being comparable across the groups with no significant differences observed in percent of successful implantations between control and treatment groups was further evident with majority of the females across groups showing normal pregnancy equal to 22 days. Also no changes in the pre-coital interval, percentage of pre and post-implantation loss in all treatment groups showed lack of dose dependent changes. From the results obtained in the present reproductive/developmental toxicity study, 3'-HPT was found to be safe at the doses of 20, 100 & 200 mg/kg bwt in Wistar rats when administered orally.

To conclude the study was performed as per OECD guideline 408, 421 respectively in rats and based on the above results, which showed no overt toxic changes in both 90 day and reproductive toxicity study, it could be suggested that a repeated oral exposure to test item 3'-hydroxypterostilbene, up to 200 mg/kg bwt/d was found to be safe and may be treated as ‘No Observed Adverse Effect Level’ (NOAEL) under the test conditions employed.

## Supporting information

S1 Fig Effect of oral administration of 3'-HPT on food consumption (average) in SD rats measured over 90 days. (A) Male SD rats (B) Female SD rats. Data in Mean ± SD (n = 10/group).
(EPS)
